# The Relation between Morphology of Maxillary Sinus after Augmentation in Three Classification Methods and Residual Bone Height: A Retrospective Study

**DOI:** 10.1155/2022/1850012

**Published:** 2022-09-29

**Authors:** Zhi Wang, Qi Jia, Heng Bo Jiang, Jianmin Han, Lidong Zou, Guangliang Niu

**Affiliations:** ^1^Second Clinical Division, School and Hospital of Stomatology, Peking University, Beijing 100101, China; ^2^Department of Dental Materials, Peking University School and Hospital of Stomatology, Beijing 100081, China; ^3^The Conversationalist Club, School of Stomatology, Shandong First Medical University, Jinan, Shandong 250117, China; ^4^Department of Oral Prosthodontics, The First Affiliated Hospital of Nanchang University, Nanchang 330006, China

## Abstract

Maxillary sinus augmentation is critical to oral implantology, particularly in some cases. The morphology of the sinus floor reflects the lifting effect to a certain extent; however, there has been limited research on the morphology after sinus augmentation. The present study aims to investigate the relationship between residual bone height (RBH) and the morphology of the sinus floor and determine whether a correlation exists between the different evaluation classifications. Maxillary sinus floor augmentation procedures were performed in 56 patients and 68 teeth using the sinus crest approach (SCA). Imaging results obtained after one year of sinus augmentation were analyzed and simultaneously classified along the coronal plane, the sagittal plane, and the biplane (coronal-sagittal). The higher the RBH, the closer the result tends to be to A, A', or type 1 (more satisfactory). There was a significant correlation between the three different evaluation classifications (*p* < 0.05). The morphology of perforation cases was involved in types C, D, C', and D'. A more satisfactory post-lifting morphology (tent type and flat type) is probably related to an optimal preoperative bone height, and an unsatisfactory post-lifting morphology is related to a low preoperative sinus floor height. The sagittal plane evaluation correlates with the coronal plane and biplane evaluation and is thus more recommended.

## 1. Introduction

Adequate bone quantity and quality are highly indispensable for successful implant therapy. The posterior edentulous maxilla presents unique challenges for implant placement. The most important among these is the presence of the maxillary sinus. Tooth extraction is often followed by maxillary sinus pneumatization and alveolar ridge resorption. The rehabilitation of the posterior maxilla depends on the amount of bone present below the sinus [1]. Implant placement and prosthetic rehabilitation of the posterior maxilla can be challenging due to maxillary alveolar atrophy after dental extractions and maxillary sinus pneumatization. Maxillary sinus floor augmentation (i.e., sinus augmentation)—described first by Boyne and James, separately by Tatum, and modified by many others—is a conventional procedure with low complication and failure rates [2]. Currently, two main sinus augmentation procedures are the osteotome technique (sinus crystal approach, SCA) and the lateral window approach. The lateral window approach, described by Tatum in 1986, is based on creating a window into the sinus (antrostomy) in the buccal bone; the bone graft material is then introduced into the sinus and placed beneath the elevated sinus membrane. This procedure allows clinicians to perform optimal sinus floor elevation and create clear and direct visualization of the operative field. Furthermore, it affords good separation of the sinus floor mucosa and a total lift. However, the surgical technique has a steep learning curve, potential risk of trauma, and high treatment costs [3, 4]. Moreover, the blood vessels in the anterior and lateral walls of the maxillary sinus can increase the risk of bleeding and limit the use of this technique to some extent [5].

**Figure 1:**
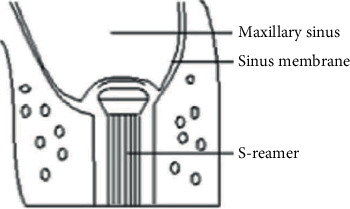
Schematic diagram of a bone disk.

The osteotome technique, described by Summers [6], is based on fracturing the maxillary sinus floor. The osteotomy procedure is initiated using a drill that stops 1-2 mm below the maxillary sinus floor. Then, an osteotome is introduced through the osteotomy. The practitioner strikes the osteotome with a mallet to fracture the bone and punch a hole into the sinus, thereby raising the sinus floor. The bone graft material is then introduced into the sinus through the osteotomy, followed by dental implant placement. Minimally invasive tools, such as the initial drill, S-reamer, stoppers, depth gauge, and bone graft equipment, are used in SCA for maxillary sinus floor augmentation [7]. Of these tools, the S-reamer is the most critical instrument for sinus floor bone grinding. The unique design enables the S-reamer to drill through the bone at a rotation speed of 1,200 rpm without tearing the sinus membrane. The reamer is pushed into the sinus cavity using an appropriate force to detach a thin layer of the inferior cortical bone and place it into the sinus. This thin layer of bone becomes a barrier between the reamer head and the sinus membrane, preventing direct contact [7]. It is called a “bone chip” and prevents membrane tearing along the S-reamer (Figure 1). The osteotome technique significantly reduces the trauma and pain associated with the open approach. Moreover, it increases stability and is associated with few complications. However, the possible lifting height is limited [3] as the residual bone height (RBH) influences the result. Furthermore, the difficulty of operation is significantly affected by the morphology of the sinus floor, and it is challenging to deal with complications such as perforation.

Therefore, maxillary sinus floor augmentation has been extensively studied to minimize trauma, yielding promising results [8]. The osteotome technique and the lateral window approach are clinically advanced techniques, and studies have confirmed the results of sinus lifting after surgery. The evaluation indicators of the effect generally focus on imaging performance, bone resorption, and marginal bone stability [9, 10]. The morphology of the sinus floor reflects the lifting effect to a certain extent. However, there has been limited research on sinus morphology after sinus floor augmentation.

This study investigated the relationship between RBH and sinus floor morphology after applying SCA for internal elevation by observing sinus floor morphology by cone beam computed tomography (CBCT). It investigated whether a correlation exists between the different evaluation classifications. The null hypothesis of this study was that there is no significant difference in the relationship between RBH and sinus floor morphology and no correlation with the different evaluation classifications (*p*=0.05).

## 2. Materials and Methods

### 2.1. Participants

**Figure 2:**
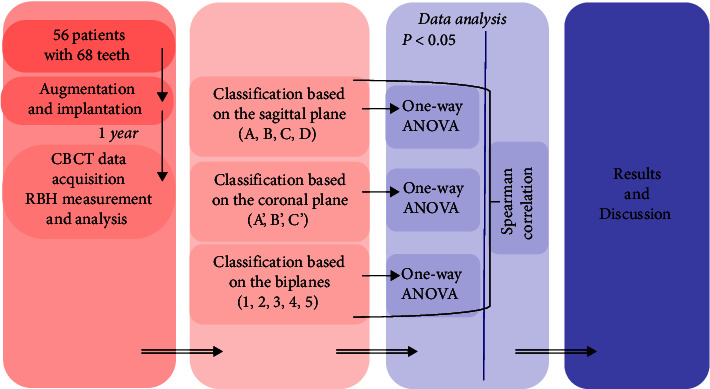
Flow chart of this study.

The study was approved by the Biomedical Ethics Committee of Peking University School of Stomatology (ethics approval number: PKUSSIRB-201837091). A total of 56 patients and 68 teeth with dentition defects in the upper posterior area treated at the second dental center, Peking University Hospital of Stomatology, from January to December 2015, were selected. All phases of the study were performed by the same unit. The patients included 31 men and 25 women aged 23–76 years (46.9 ± 10.8 years). The RBH of the sinus floor was 4–9 mm, representing the distance between the crest of the central point of the missing tooth area and the bottom of the sinus.  Figure 2 shows the flow chart of this study. Cone beam computed tomography (CBCT; DCTPRO, VATECH, Gyeonggi-do, South Korea) was used for all radiographic examinations. The measurement software package used was CBCT (Ez3D 2009 v.1.2.4.1, VATECH, Gyeonggi-do, South Korea).  Table 1 shows the inclusion and exclusion criteria of this study.

### 2.2. Interventions and Outcomes

The study used minimally invasive tools (SCA KIT, Neobiotech, Seoul, South Korea) for sinus augmentation. The operation was performed by two experienced senior clinicians. Prophylactic antibiotic treatment was administered (1 g of amoxicillin 1 h before the procedure). The patient was asked to rinse with a mouthwash containing chlorhexidine gluconate 0.2% solution for one minute. Surgery commenced with the injection of articaine hydrochloride and epinephrine tartrate for anesthesia and a crestal incision, without vertical extensions, along the maxillary ridge. A full-thickness mucoperiosteal flap was elevated to expose the alveolar ridge, implant sites were prepared, and 1 mm of the sinus bottom bone was preserved. An S-reamer with a suitable diameter and stopper was chosen depending on the specific case to remove the remaining bone of the sinus floor. Appropriate pressure was applied to push 1 mm into the sinus. Loss of the sensation of resistance occurred on complete removal of the sinus floor. Then, an inspection instrument with an identical stopper was used to examine the sidewall of the prepared cavity. If decortication of the sinus floor was not achieved, a longer stopper was used to grind the remaining bone; when finished, nasal aspiration was performed to confirm whether the sinus floor mucosa was perforated.

It was confirmed that the sinus floor mucosa was intact and the lifting height exceeded 2 mm. Then, implants were placed with 0.25 g of Geistlich Bio-Oss (Geistlich Pharma, Lucerne, Switzerland). Among the 68 implants, five were 8–8.5 mm implants and the rest were 10 mm implants of diameter 4.5/4.8 mm. Sixty-five implants were placed with torque values >15 N° cm and the one-stage procedure; three implants were placed with torque values <15 N° cm and the submerged healing protocol. Routine preventive antibiotic treatment was administered after surgery: amoxicillin 0.5 g thrice daily and tinidazole 1 g once daily. The patients were asked to rinse with 0.02% chlorhexidine for two minutes thrice daily. Furthermore, they received antibacterial mouth rinse, systemic antibiotics, nasal drops, and inhalants for 7–10 days after the operation. Sutures were removed 7–10 days after surgery. All patients were instructed on appropriate oral hygiene measures immediately after surgery and reinstructed after the uncovering procedure and during follow-up sessions.  Table 2 shows the study protocol.

### 2.3. Evaluation and Statistical Methods

**Figure 3:**
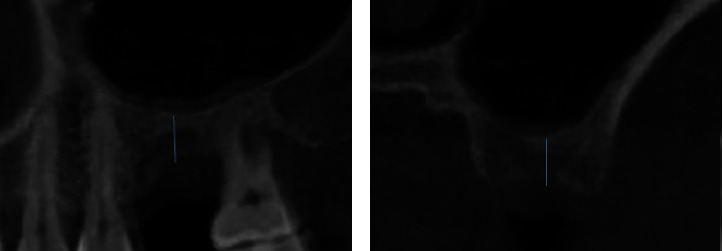
Residual bone height (RBH) based on the (a) sagittal plane and (b) coronal plane.

RBH was measured before the operation (Figure 3). One-year follow-up CBCT data were used for morphological analysis of the sagittal and coronal planes. Origin Pro (v. 2022, Massachusetts, USA) was used in this study. One-way ANOVA was performed for RBH data comparison between the types, while the confidence interval was 95%. (*p* < 0.05) The correlation between various evaluation methods was analyzed using Spearman correlation analysis, and significance was redetermined as *p* < 0.05 for all tests performed.

## 3. Results

All the implants were clinically successful. They received postoperative follow-ups, and upper arch restoration was completed 3–6 months after surgery. No patients were lost in this study.

### 3.1. Classification

According to Song et al. [11], the types of bone graft morphology after minimally invasive maxillary sinus floor augmentation can be divided into the following .

#### 3.1.1. Classification Based on the Sagittal Plane

**Figure 4:**
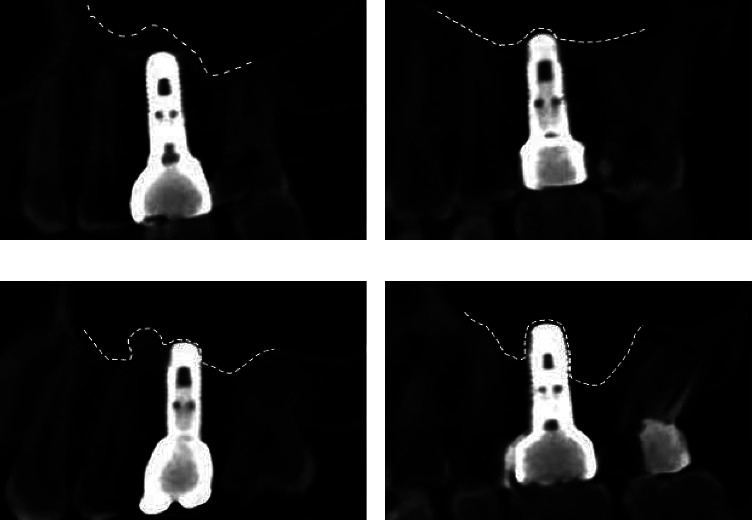
Classification based on the sagittal plane. (a) Type A: tent type; (b) Type B: flat type; (c) Type C: one-sided dehiscence type; (d) Type D: two-sided dehiscence type.

  Type A: *Tent Type*. The images show bone or bone graft material around the implant, and the implant does not come into contact with the sinus floor mucosa (Figure 4)
  Type B: *Flat Type*. Only the tip of the implant is in contact with the sinus floor mucosa
  Type C: *One-Sided Dehiscence Type*. The mesial or distal surfaces of the implant and the tip of the implant are in contact with the sinus floor mucosa
  Type D: *Two-Sided Dehiscence Type*. The proximal and distal surfaces of the implant and the tip of the implant are in contact with the sinus floor mucosa

#### 3.1.2. Classification Based on the Coronal Plane

  Type A′: *Tent Type.* The images show bone or bone graft material around the implant, and the implant does not come into contact with the sinus floor mucosa
  Type B': *Flat Type.* Only the tip of the implant is in contact with the sinus floor mucosa
  Type C′: *One-Sided Dehiscence Type*. The buccal or lingual side of the implant and the tip of the implant are in contact with the sinus floor mucosa
  Type D': *Two-Sided Dehiscence Type*. The buccal and lingual sides and the implant tip are in contact with the sinus floor mucosa

#### 3.1.3. Classification Based on Biplanes

  Type 1: *Tent Type.* The images show bone or bone graft material around the implant, and the implant does not come into contact with the sinus floor mucosa
  Type 2: *Flat Type*. Only the tip of the implant is in contact with the sinus floor mucosa
  Type 3: *One-Sided Dehiscence Type*. In addition to the tip of the implant, one of the mesial, distal, buccal, or lingual surfaces is in contact with the sinus floor mucosa
  Type 4: *Two-Sided Dehiscence Type*. In addition to the tip of the implant, two mesial, distal, buccal, or lingual surfaces are in contact with the sinus floor mucosa
  Type 5: *Three-Sided Dehiscence Type*. In addition to the tip of the implant, three mesial, distal, buccal, or lingual surfaces are in contact with the sinus floor mucosa
  Type 6: *Four-Sided Dehiscence Type*. In addition to the tip of the implant, the mesial, distal, buccal, and lingual surfaces are all in contact with the sinus floor mucosa

### 3.2. The Morphology Based on the Sagittal Plane

**Figure 5:**
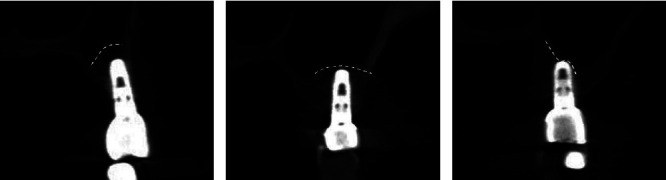
Classification based on the sagittal plane. (a) Type A': tent type; (b) Type B': flat type; (c) Type C': one-sided dehiscence type.

**Figure 6:**
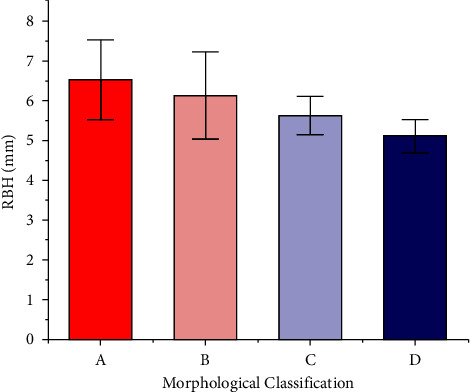
The mean RBH of different morphological classifications based on the sagittal plane (*p* < 0.05; power = 0.83). (a) Type A: tent type; (b) Type B: flat type; (c) Type C: one-sided dehiscence type; (d) Type D: two-sided dehiscence type.

There were 46 cases of Type A, two cases of Type B, four of Type C, and six of Type D (Figure 5) (Table 3 and Figure 6). The mean RBH of Types A, B, C, and D cases was 6.53 mm, 6.13 mm, 5.63 mm, and 5.10 mm, respectively (*p* < 0.01). Figure 6 shows that the mean value of RBH gradually decreased as the morphology deteriorated on the sagittal plane.

### 3.3. The Morphology Based on the Coronal Plane

**Figure 7:**
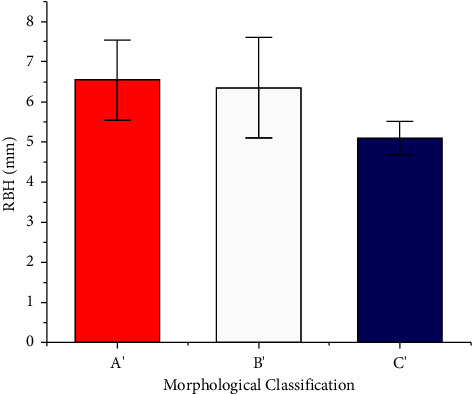
The mean RBH of different morphological classifications based on the coronal plane (*p* < 0.05, power = 0.63). (a) Type A': tent type; (b) Type B': flat type; (c) Type C': one-sided dehiscence type.

There were 28 cases of Type A', 34 cases of Type B', six of Type C', and none of Type D' (Table 4 and Figure 7). The mean RBH of Types A', B', and C' cases was 6.54 mm, 6.35 mm, and 5.33 mm, respectively (*p* < 0.05). The mean value of RBH gradually decreased as the morphology deteriorated on the coronal plane, especially in Type C'.

### 3.4. The Morphology Based on the Biplanes

**Figure 8:**
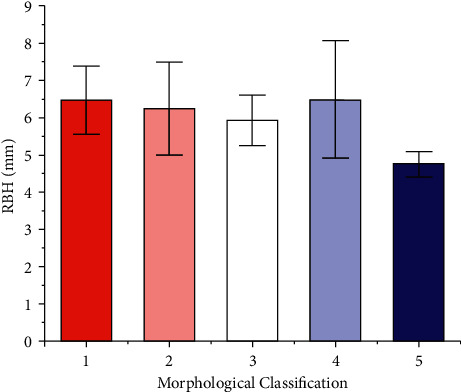
The mean RBH of different morphological classifications based on biplanes (*p* > 0.05, power = 0.47). (1) Type 1: tent type; (2) Type 2: flat type; (3) Type 3: one-sided dehiscence type; (4) Type 4: two-sided dehiscence type; (5) Type 5: three-sided dehiscence type.

There were 36 cases of Type 1, 16 cases of Type 2, nine of Type 3, five of Type 4, and two cases of Type 5 (Table 5 and Figure 8). The mean RBH of Types 1, 2, 3, 4, and 5 cases was 6.47 mm, 6.25 mm, 5.93 mm, 6.50 mm, and 4.75 mm, respectively (*p* > 0.05). Except for Type 4 (6.50 ± 1.58 mm), the mean value of RBH gradually decreased as the morphology deteriorated on the biplanes.

### 3.5. Correlation between Categories

**Figure 9:**
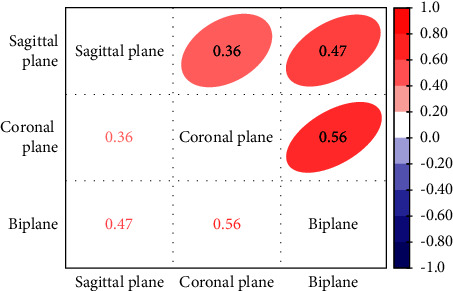
Correlation heatmap between evaluation methods. ^*∗*^Positive correlations are represented in red, and negative correlations are represented in blue. Stronger correlations are represented by darker colors.

Spearman correlation analysis was performed for the results obtained with the three classification methods (Table 6 and Figure 9). According to the definition methods of Chan (Medicine) [12], the results were as follows: there was a “Fair” correlation (0.36) between the sagittal classification and coronal plane classification, “Fair” correlation (0.47) between sagittal classification and biplane classification, and “Moderate” correlation (0.56) between coronal plane classification and biplane classification.

## 4. Discussion

This study proposed a new method to evaluate the internal structure and volume changes after sinus augmentation. Reports show several techniques for the assessment of augmented bone changes. We focused on the morphological analysis of CBCT images. Previous studies have used bone graft volume with CBCT and specific software; for example, Aktuna Belgin et al. [13] used CBCT and Mimics software to assess the bone volume of the maxillary sinus. An analysis of graft material using CBCT provided both linear measurements and 3-D evaluations [14, 15]. However, 3-D evaluation using CBCT requires extensive manual work, making it a laboratory application instead of a clinical technique [16, 17]. There are no standardized methods for volume measurement using serial CBCT images after sinus augmentation. Accurate measurement of the graft material through 3-D analysis software can be challenging due to the varying degrees of opacity at the bone boundaries [18, 19] and the unclear cortical lining of the sinus floor. Second, the patient's position cannot be replicated for each serial CBCT image capture. Third, the gray value is inaccurate for each measurement of CBCT images. Lastly, reliable volume measurement and standardized bone quality evaluation are technically challenging due to errors in manual segmentation [20]. However, the measurements based on bone graft volume are not the best evaluation methods, and the classification of bone volume is inaccurate. Therefore, we aimed to determine the extent to which the root tip area of the implant was surrounded by bone.

**Figure 10:**
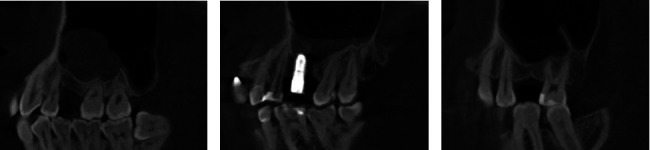
Possible reasons for unsatisfactory morphologies. (a) cyst; (b) sinus membrane perforation; (c) oblique sinus floor.

In this study, Types A, B, A', B', 1, and 2 were satisfactory morphologies and the remaining were unsatisfactory. The possible reasons for unsatisfactory morphologies are as follows: (1) The RBH is <5 mm, and the lifting height is limited. There were four cases with RBH <5 mm. (2) The cyst increased pressure in the sinus and posed difficulty for a sinus lift (Figure 10(a)). (3) Sinus membrane perforation was observed (Figure 10(b)). (4) An oblique sinus floor existed, and the peeling off one side was insufficient (Figure 10(c)). (5) Sinus augmentation was done without bone grafting.

The difference between the coronal and sagittal plane classification is the absence of type D in the coronal plane. In this regard, the findings of our study are consistent with a previous study [11]. In evaluations performed on the coronal plane, the cases were divided into Types A (tent type), B (flat type), and C (one-sided dehiscence type). However, our study included cases that met the criteria for Type D in evaluations based on the sagittal plane, probably because the mesial-distal distance of the maxillary sinus in the sagittal plane was relatively large, allowing observation of changes in the bone graft morphology in the sagittal plane abundantly. The transverse distance on the coronal plane was smaller; therefore, it was hard to observe the difference between Types C and D. The correlation between the classifications was significant, and the biplane classification was the most detailed but more complicated.

Tables 3–5 show significantly different morphologies under the different mean RBH, consistent with the results of the previous study. Reportedly, the survival rate of implants increases with an increase in remaining alveolar ridge height. The implant survival rate is ≥96% when the elevation of the front bone height is >5 mm, but the survival rate is 85.7% when it is <4 mm [21]. It is noteworthy that the minimum RBH of Types A and B (satisfactory morphologies) on the sagittal is not lower than that of Types C and D (unsatisfactory morphologies) (Table 3). The same goes for the coronal plane and biplanes (Tables 4 and 5). Thus, unsatisfactory morphology usually comes from low RBH but not all low RBH results in unsatisfactory morphology; it may be affected by the quality of healing (such as the peri-implant microbiota) [22]. Overall, the trend of RBH declined as the morphology got worse.

Correlations were also observed between the coronal plane classification and the biplane classification and between the sagittal plane classification and biplane classification, suggesting that one of the planes can be used in the evaluation. Some studies have favored classification based on coronal planes [11], although the specific reasons underlying these preferences are unknown. Based on the findings of this study, the authors recommend the sagittal plane because the maxillary sinus has a wider field of view in the sagittal plane, yielding richer information and allowing better evaluation of the lifting effect.

The most common intraoperative complication of sinus floor elevation is perforation of the Schneiderian membrane, which may yield unsatisfactory morphologies. Meanwhile, some studies have reported that implant penetration into the sinus membrane caused by perforation does not affect long-term implant stability. The perforation area caused by internal lifting is relatively small, and a smaller perforation can be folded on its own without implant loss [23]; however, this process affects bone graft morphology. This article included four cases of perforation, classified in the sagittal and coronal positions.  Table 7 shows that the morphological classification of sagittal and coronal morphologies after perforation is not ideal.

Many studies have shown an improved bone volume of the sinus floor without bone grafting to achieve osteogenesis [24]. Implants without bone grafting with simultaneous osteotome sinus floor elevation (OSFE) can increase bone height in the surrounding sinuses of the implant. Furthermore, studies confirm that new bone formation can occur in the sinuses without bone grafting with implant osseointegration. Lai et al. [25] reported a cumulative survival rate of 95.71% and concluded that OSFE with and without bone grafts yields similar results. A similar cumulative survival rate was seen by Pjetursson et al. [26]. Winter et al. placed 58 implants using sinus/alveolar crest tenting and reported a 91% survival rate [27].

However, others have shown that the osteogenic effect after bone grafting remains better than the effect without bone grafting. Trinh et al. [28] showed that the acemannan-treated group had an average EsBG (Endo-sinus bone gain) of 2.46 ± 0.4 and 3.26 ± 0.3 mm, at three and six months after surgery, respectively, while that of the control was 1.16 ± 0.3 and 1.66 ± 0.3 mm, respectively. Some studies have indicated that implant placement into the sinus without graft materials can stimulate new bone formation in the sinus cavity [29]. Specifically, blood cells induce new bone formation by stimulating bone precursor cells to differentiate into osteoclasts, and these activated osteoclasts activate other bone-forming osteoclasts to produce bone [30]. According to Silva et al. [24], the results without bone graft material in the maxillary sinus are similar to those using bone graft material; however, it may result in space-maintenance problems. Without bone graft material, the space-maintenance ability of blood-clot formation alone might be diminished [31]. Thus, it is necessary to determine the amount and morphology of bone formation, especially related to implant length. It is also crucial to evaluate maxillary sinus morphology [11]. The apical area of the implant without a bone graft is covered by mucosa [32, 33]. Without the addition of bone graft material, the stability of the space underneath the sinus membrane is primarily dependent on the implant apex and the associated blood clot after surgery. Early clot breakdown during the healing process can result in membrane collapse and limit the amount of bone formation. The bone graft results in a higher mean bone gain percentage compared to no bone graft [34].

A previous study found bone defects in approximately five implants (5/46; 10.9%), of which four were in the palatal region and one in the buccal region [11], which may occur when the membrane falls onto the implant due to the air pressure in the maxillary sinus, thus inhibiting blood clot stability.

Because of the patients lost, there were only 56 patients and 68 teeth in this study. Moreover, because of the uneven proportion of the types in the three classification methods, the number of types in one classification was unbalanced, which could have produced an error in the results, leading to low power. This study could provide a reference to dentists for risk prevention but could not calculate the specific prognosis with specific RBH, a limitation of the retrospective study [35].

Future studies should evaluate how factors that affect bone graft morphology can also affect long-term implant stability. Further research should also determine if the sinus morphology and the long-term implant stability are correlated. Therefore, future studies can perform long-term follow-ups to evaluate the survival rate of the sinus augmentation and implant.

## 5. Conclusion

A more satisfactory post-lifting morphology (tent type; flat type) is probably related to an optimal preoperative bone height, and an unsatisfactory post-lifting morphology is related to a low preoperative sinus floor height. The sagittal plane evaluation is correlated with the coronal plane and biplane evaluation and is recommended for evaluating bone morphology after maxillary sinus augmentation.

## Figures and Tables

**Table 1:** Inclusion and exclusion criteria.

Inclusion criteria
Age ≥18 years
Need of sinus floor augmentation
A residual bone height of approximately ≥4 mm as observed by radiographic examination
Passage of at least three months after tooth loss in the intended sinus augmentation location
Healthy sinus as determined by radiographic examination
Periodontal health stable
No general health contraindications

Exclusion criteria
General contraindications to implant surgery
A history of radiation therapy in the head and neck area
A history of treatment or being under treatment with intravenous amino-bisphosphonates
Poor oral hygiene and motivation

**Table 2:** Protocol designed for the study.

Appointment	Procedures
1 h before the procedure	Prophylactic antibiotic treatment (1 g of amoxicillin 1 h before the procedure).
During procedure	Rinse with a mouthwash containing chlorhexidine gluconate 0.2% solution for 1 minute.
	Injection of articaine hydrochloride and epinephrine tartrate for anesthesia.
	Create a crystal incision without vertical extensions along the maxillary ridge.
	Exposure of the alveolar ridge.
	Preparation of the implant sites.
	Preservation of the sinus bottom bone (1 mm).
	An S-reamer with a suitable diameter and stopper was chosen for specific cases to remove the remaining bone from the sinus floor. During this process, appropriate pressure was applied to push into the sinus by 1 mm with the reamer.
	A sense of loss (completely remove of sinus floor)—check along the sidewall of the prepared cavity.
	Or, remain on the floor—grinding of the remaining bone with a longer stopper.
	Nasal aspiration—to check whether the sinus floor mucosa was perforated.
	Implant placement.
After surgery	Routine preventive antibiotic treatment.
	An antibacterial mouth rinse, systemic antibiotics, nasal drops, and inhalants.
One year after the procedure	CBCT data acquisition.

**Table 3:** The RBH values are compared between of different morphological classifications based on the sagittal plane.

Morphology	Mean RBH (mm)	SD (mm)	Max (mm)	Min (mm)	Median (mm)
*A*	6.53	1.00	8.50	4.50	6.75
*B*	6.13	1.10	8.00	5.00	5.75
*C*	5.63	0.48	6.00	5.00	5.75
*D*	5.10	0.42	5.50	4.50	5.00

*p* < 0.01 (power = 0.83).

**Table 4:** The RBH values were compared between different morphological classifications based on the coronal plane.

Morphology	Mean RBH (mm)	SD (mm)	Max (mm)	Min (mm)	Median (mm)
*A*	6.54	1.00	8.50	4.50	6.50
*B*	6.35	1.26	9.00	4.00	6.00
*C*	5.33	0.42	5.50	4.50	5.00

*p* > 0.05 (power = 0.63).

**Table 5:** The RBH values were compared between different morphological classifications based on the biplanes.

Morphology	Mean RBH (mm)	SD (mm)	Max (mm)	Min (mm)	Median (mm)
1	6.47	0.92	8.00	4.50	6.50
2	6.25	1.25	8.00	4.50	6.00
3	5.93	0.68	7.00	5.00	5.75
4	6.50	1.58	9.00	5.00	6.00
5	4.75	0.35	5.00	4.50	4.75

*p* > 0.05 (power = 0.47).

**Table 6:** The Spearman correlation between evaluation methods.

Evaluation methods	Spearman correlation	*p* value	Type (Chan YH-medicine)
Sagittal plane-Coronal plane	0.36	0.002	Fair
Sagittal plane-Biplane	0.47	<0.001	Fair
Coronal plane-Biplane	0.56	<0.001	Moderate

**Table 7:** Morphology types in the perforation cases.

Case	Sagittal morphology	Coronal morphology
1	*C*	*C*′
2	*D*	*C*′
3	*D*	*C*′
4	*D*	*C*′

## Data Availability

The data used to support the findings of this study are included in this article.
